# MUG: A mutation overview of GPCR subfamily A17 receptors

**DOI:** 10.1016/j.csbj.2022.12.031

**Published:** 2022-12-21

**Authors:** Ana B. Caniceiro, Beatriz Bueschbell, Carlos A.V. Barreto, António J. Preto, Irina S. Moreira

**Affiliations:** a CNC - Center for Neuroscience and Cell Biology, Center for Innovative Biomedicine and Biotechnology, University of Coimbra, Coimbra, Portugal; b PhD in Biosciences, Department of Life Sciences, University of Coimbra, Calçada Martim de Freitas, 3000-456 Coimbra, Portugal; c PhD Programme in Experimental Biology and Biomedicine, Institute for Interdisciplinary Research (IIIUC), University of Coimbra, Casa Costa Alemão, 3030-789 Coimbra, Portugal; d Department of Life Sciences, University of Coimbra, Calçada Martim de Freitas, 3000-456 Coimbra, Portugal

**Keywords:** GPCRs, G protein-coupled receptors, GEFs, GTP-exchange factors, FDA, Food and Drug Administration, LOF, loss-of-function, GOF, gain-of-function, MUG, mutations understanding GPCRs, TM, transmembrane, ECL, extracellular loop, ICL, intracellular loop, G protein-coupled receptors, GPCR subfamily A17, Natural variants, Database, Neurodegenerative diseases

## Abstract

G protein-coupled receptors (GPCRs) mediate several signaling pathways through a general mechanism that involves their activation, upholding a chain of events that lead to the release of molecules responsible for cytoplasmic action and further regulation. These physiological functions can be severely altered by mutations in GPCR genes. GPCRs subfamily A17 (dopamine, serotonin, adrenergic and trace amine receptors) are directly related with neurodegenerative diseases, and as such it is crucial to explore known mutations on these systems and their impact in structure and function. A comprehensive and detailed computational framework - MUG (Mutations Understanding GPCRs) - was constructed, illustrating key reported mutations and their effect on receptors of the subfamily A17 of GPCRs. We explored the type of mutations occurring overall and in the different families of subfamily A17, as well their localization within the receptor and potential effects on receptor functionality. The mutated residues were further analyzed considering their pathogenicity. The results reveal a high diversity of mutations in the GPCR subfamily A17 structures, drawing attention to the considerable number of mutations in conserved residues and domains. Mutated residues were typically hydrophobic residues enriched at the ligand binding pocket and known activating microdomains, which may lead to disruption of receptor function. MUG as an interactive web application is available for the management and visualization of this dataset. We expect that this interactive database helps the exploration of GPCR mutations, their influence, and their familywise and receptor-specific effects, constituting the first step in elucidating their structures and molecules at the atomic level.

## Highlights

• GPCRs subfamily A17 are associated with neurogenerative diseases with genetic errors as a major cause for such pathologies
• MUG is a detailed database, which illustrates the known mutations and their effects on receptors of this key GPCR subfamily.
• MUG allows a functional understanding of mutations by providing their structural location and potential pathogenicity.
• MUG can guide the discovery of clinically relevant mutations and the clarification of the potential deregulation.

## Graphical Abstract

**Figure:**
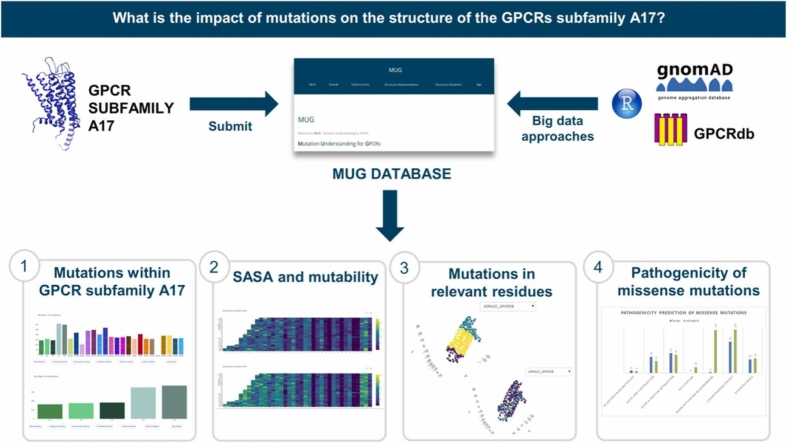

## 1 Introduction

More than 800 genes, 4.1%− 4.3% of the human genome, code for G protein-coupled receptors (GPCRs), making them the largest family of membrane proteins [1], [2], [3]. Members of the GPCR superfamily are involved in almost every physiological function, ranging from neurotransmission, hormone regulation, and metabolite-, odor- and ion-signaling as well as the signal transduction of mechanical forces and cell-cell and cell-matrix interactions [3], [4], [5], [6], [7], [8], [9], [10], [11], [12]. The function of GPCRs can be described as the signal-driven conformational change of a receptor, leading to activation of an effect in the downstream signaling cascade inside the cell, resulting in either loss or recruitment of proteins, change of ion concentration or modification of enzymatic activity [3]. Furthermore, GPCRs can also be seen as GTP-exchange factors (GEFs), and activation of a receptor is known to lead to the exchange of GDP to GTP in the alpha subunit of associated heterotrimeric G proteins [3]. Since GPCRs can mediate a wide array of signals, they are also targeted by over 35% of United States Food and Drug Administration (FDA)-approved drugs [13], [14]. In 2016, of a total of 1286 approved FDA drugs, 460 were GPCR-targeting drugs [15].

In addition to the many physiological functions, GPCRs were reported to regulate pathophysiological states and were associated with several severe diseases [16], [17]. The root cause of such pathologies is mostly genetic errors, which alter the normal function of the receptor [3]. The most frequent mutations that alter the function of GPCRs are generally classified according to an inactivation of their GEF activity, called loss-of-function (LOF), or with a ligand-independent activation of this GEF activity, called gain-of-function (GOF). However, a simple distinction between LOF and GOF does not fully reflect the variety of disease-causing mutations, due to the complexity of the GPCR signaling process. Over 2350 mutations in 55 GPCR genes have been directly linked to 66 human monogenic diseases, making the contribution of GPCR genes to monogenic human diseases approximately 18% [3]. In addition, it is also known that a single GPCR gene can cause different diseases, such as hyper- and hypothyroidism, due to inactivating and activating mutations in the thyrotropin (TSH) receptor [3]. Most mutations are missense mutations (68%), small insertions/deletions (16%), nonsense (stop) mutations (7%), gross deletions/rearrangements (6%), and splice-site mutations (3%) [3], [18]. Deletions or insertions of amino acids in the coding sequence and nonsense mutations lead to the nonfunctioning of a receptor. Aside from these types of mutations, the diverse spectrum of point mutations, designated by missense mutations, can lead to a clear modification of the functionality of a receptor. If such single events spread across the populations and reach a frequency over 1%, they are defined as a natural variant or allele [19].

The relationship between protein structure and function is a central issue, i.e., a replacement or alteration in the amino acid sequence can bring changes in the folding and stability of the protein, interaction with other molecules, protein activity and function, and drug susceptibility [20]. Location of the mutation can promote different effects on receptor activity, such as ligand binding and the ability to bind to G proteins and arrestin proteins, as well as receptor trafficking to the cell surface. Thus, it is crucial to analyze the type of mutations that occur in residues involved in the mechanism of action of GPCRs.

In our study, we developed a database, MUG (mutations understanding GPCRs), which provides an overview of already described mutations and their effect on receptors of subfamily A17. Similar to all class A GPCRs, the members of subfamily A17 share a common architecture of seven transmembrane helices (7 TMs) connected through three extracellular (ECL1–3) and three intracellular loops (ICL1–3) as well as an extracellular N-terminus and an intracellular C-terminus [21], [22]. The subfamily A17 comprises receptors that bind to biogenic amines [23], including dopamine receptors (D_1–5_R), serotonin receptors (5-HT_2A-C_R, 5-HT_6_R), trace amine receptors (TA_1–3_R, TA_5–6_R, TA_8–9_R), and adrenergic receptors (α_1A/1B/1D_-adrenoceptor, α_2A/2B/2 C_-adrenoceptor, β_1/2/3_-adrenoceptor). Although this subfamily is known to be the unique subfamily directly associated with neurodegenerative diseases [24], [25], [26], [27], [28], is still poorly studied. As such, these receptors are good study subjects to better characterize and understand reported mutations and their impact on structure and function. We located the mutations in receptor structure and analyzed the type of mutations occurring overall and in the different families of subfamily A17 as well as what effects they have on receptor functionality. Moreover, we depicted the mutated enriched positions in subfamily A17. MUG provides a straightforward approach to analyze and characterize GPCR families by their mutational landscape.

## 2 Materials and methods

### 2.1 Data acquisition and filtering

Natural sequence variations from functionally annotated members of GPCR subfamily A17 (dopamine, serotonin, adrenergic and trace amine receptors) were downloaded from the Genome Aggregation Database (gnomAD v2.1.1) [29]. GnomAD is a database with the purpose of aggregating and harmonizing both exome and genome sequencing data from a wide range of large-scale sequencing projects all over the world and making summary data available for the wider scientific community [29]. GnomAD allows to interpret human biology using large-scale genomic datasets Furthermore, gnomAD enable a wide range of scientific applications and is an added value in the mutation analysis [30]. Only mutations that are part of the coding region were selected, including missense, synonymous, frameshift, insertion, deletion, stop gained, start lost and stop lost variants.

Sequences of all GPCR subfamily A17 receptors were obtained from the GPCR database (GPCRdb) [31]. Instead of the Generic Numbering followed in GPCRdb, we used the well-established Ballesteros-Weinstein (BW) numeration [32] to create a uniform and comparable nomenclature. In-house scripts were employed to mine sequence variations and alignment of mutations to sequences. Pointed mutations were organized according to their position on the sequence and structure of the various receptors. Data were filtered to remove duplicates and sequence conflicts with GPCRdb [31] subfamily A17 sequences.

**Fig. 1:**
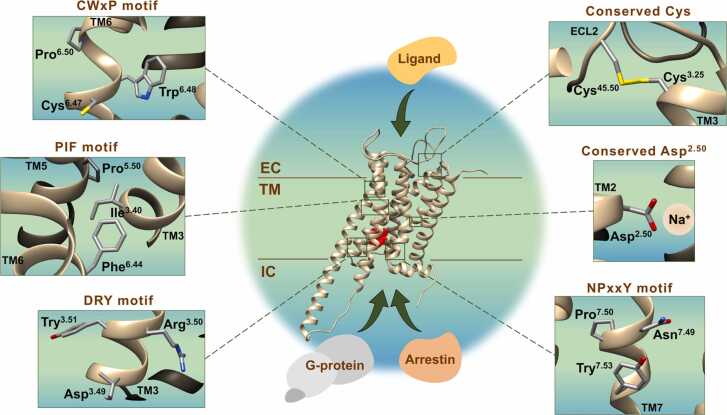
Structural visualization of the inactive D_1_R in a schematic lipid membrane. Close-ups were used to identify and locate relevant residues, including ligand binding site, allosteric binding site, known activating microdomains (DRY, PIF, CWxP, and NPxxY), key cysteines, GPCR-G protein interacting site, GPCR-Arrestin interacting site and other residues. Ligand binding site, GPCR-G protein and GPCR-Arrestin interacting sites were indicated by green arrows. Allosteric binding site was indicated by a solid red surface.

Known mutations were divided into the following groups based on their location: i) ligand binding pocket, ii) allosteric binding pocket, iii) known activating microdomains, iv) key cysteine residues, v) GPCR-G protein interaction, vi) GPCR-Arrestin interaction, and vii) other relevant residues (Fig. 1). The data collected for each GPCR subfamily A17 receptor were imported and processed using R language (Version 4.1.0), and the R studio (Version 1.4.1717) [33].

### 2.2 GPCRs models and homology modeling

Inactive GPCR models and structures were downloaded from GPCRdb [31], [34], with the exception of D_5_R and α_2B_-adrenoceptor. As these two receptor three-dimensional structures (3D) were not available in the database at the beginning of the project, they were subjected to homology modeling for which the MODELLER package (Version 10.1) [35] was used. This software allows the construction of 3D protein models from proteins with known structures that are used as templates and that should share a high sequence homology (at least 25%) with the modeled structure [36]. The inactive D_1_R structure from GPCRdb (PDB code 4GBR, 60% sequence similarity) [37] was chosen as the template for the D_5_R model. The inactive α_2A_-adrenoceptor structure from GPCRdb (PDB code 6KUY, 52% sequence similarity) [38] was chosen as the template for the α_2B_-adrenoceptor model. The sequence alignment between D_1_R and D_5_R models and α_2A_-adrenoceptor and α_2B_-adrenoceptor models was made through the “structure-based alignments'' tool from GPCRdb. TMs and disulfide bonds were further defined for model construction. One hundred models were created for each receptor sequence, and the 10 best models from each receptor were selected using the discrete optimized protein energy score (DOPE score) [39], MODELLER objective function [40] and ProSA-web server Z-score [41]. The top three models were then visually inspected using PYMOL (Version 2.5.1) software [42]. Furthermore, AlphaFold models for GPCR subfamily A17 were also downloaded from the AlphaFold Database [43]. AlphaFold was developed by DeepMind and was shown to predict 3D models of protein structures from their amino acid sequence with a high accuracy [44]. The AlphaFold models were visually compared to GPCRdb models in the MUG database.

### 2.3 Structural features of GPCR models

From the structures of the diverse GPCRs, the prediction of membrane orientation of each receptor and, consequently, the prediction of which residues belong to the extracellular, transmembrane and intracellular domains were made using orientations of proteins in membranes (OPM) database [45]. The OPM database provides the protein structure within the lipid bilayer [45].

The solvent-accessible surface area (SASA) of all residues of each GPCR model was also calculated using visual molecular dynamics (VMD) software [46]. Furthermore, a definition of surface and interior regions was also established according to the individual residue value of the relative accessible surface area (rASA). In agreement with Miller et al.’s procedure [47], rASAs were calculated by normalizing the absolute SASA value of each residue by its value in a Gly-X-Gly peptide. In general, a residue is considered buried if its rASA is below 25% [48]. Interface regions (extracellular interface, intracellular interface, and surface) were also further split according to the functional role into ligand binding pocket, allosteric binding pocket, GPCR-G-protein interaction, and GPCR-Arr interaction.

### 2.4 Pathogenicity prediction

Pathogenic prediction tools perform a prediction and evaluation of the effect of amino acid substitutions on protein structure or function. These tools present a pathogenicity prediction based on localization within protein, biochemical properties of mutant and wild-type residues, conservation among species, and potential impact of the variation on mRNA [49]. Variants were analysed using the following pathogenicity prediction tools: Functional Analysis through Hidden Markov Models (FATHMM) [50], Protein Variation Effect Analyzer (PROVEAN) [51], Polymorphism Phenotyping v2 (PolyPhen-2) [52], Protein Analysis Through Evolutionary Relationships (PANTHER) [53], MutaFrame [54], SNAP2 [55], [56], SNPsGO [57], SuSPect [58], and Sorting Intolerant From Tolerant (SIFT) [59].

### 2.5 Statistics Treatment

The statistical analysis of the data was performed in Rstudio (Version 1.4.1717) [33]. P-values were calculated with one-way ANOVA (p < 0.05). For statistics related to amino acid exchange, sets of amino acids were split according to hydrophilic and hydrophobic potential as (i) hydrophobic residues - Ala, Ile, Leu, Met, Phe, Trp, Tyr, Val, Gly, Pro, (ii) polar residues - Ser, Thr, Asn, Gln, Cys, (iii) negatively charged residues - Asp, Glu, (iv) positively charged residues - Arg, His, Lys. The data analysis was performed using the *tidyverse* package (version 1.3.1), specifically the included packages *dplyr* for data manipulation and *ggplot2* for data visualization [60].

### 2.6 Webserver implementation

The webserver, available at http://moreiralab.com/resources/mug, was constructed using the Flask web framework with Python deployment. Data were processed using several Python packages integrated within in-house developed code. Plots were constructed using the *Plotly* package [61]. The webserver covers and extends the work described in the manuscript by highlighting several sections with dynamic plotting:a) “MUG” - the landing page, presenting the work in parallel to the present manuscript.

b) “Overall” - a page displaying broad information on the dataset, such as i) overall comparisons of mutation types according to receptor family; ii) mutation per amino acid type; iii) a broad display of the mutations table.

c) “Substructures” - informational close-ups according to GPCR substructure: i) SASA; ii) rASA; iii) missense, iv) synonymous, v) frameshift, vi) in-frame insertion, vii) in-frame deletion mutations per amino acid; viii) structural and ix) interface regions, as well as x) relevant residues.

d) “Structures Representations” - displaying three-dimensional display of the receptor structures: i) in comparison with AlphaFold; ii) displaying SASA and iii) rASA; showing iv) domain structure prediction, v) structural region display, vi) Interface prediction, vii) relevant residues prediction.

In section e) “Structures Mutations” there are also 3D representations of the receptors in which are highlighted i) synonymous, ii) missense, iii) in-frame insertion, iv) in-frame deletion and v) frameshift mutations.

## 3 Results and discussion

### 3.1 Database description

**Fig. 2:**
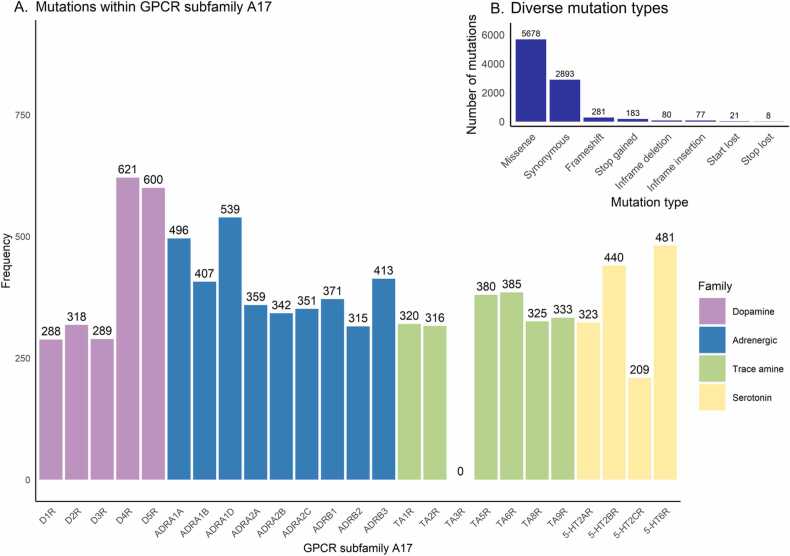
General analysis of mutation frequency over the GPCR subfamily A17 database. **A.** Total number of mutations in each receptor. **B.** Number of mutations sorted by type.

The MUG database represents all the nucleotide changes described in the gnomAD database [29] for the GPCR subfamily A17 and their functional consequences. The database consists of 25 receptors and a total of 9221 mutations (Fig. 2A). D_4_R, D_5_R and α_1D_-adrenoceptor are the three receptors with the most mutations described, with 621, 600 and 539 mutations, respectively. On the other hand, the lowest variation rates correspond to the TA_3_R receptor, which has so far, no registered nucleotide changes, only noncoding transcript exon variants. The most common mutation type in the MUG database corresponds to missense (61.6%), followed by synonymous (31.4%), frameshift (3.1%), stop gained (1.9%), in-frame deletion (0.9%), in-frame insertion (0.8%), start lost (0.2%) and stop lost (0.1%) (Fig. 2B). An overview of the database is provided and analyzed in the “Overall” section of the MUG database website.

### 3.2 Mutation distribution over domains

**Fig. 3:**
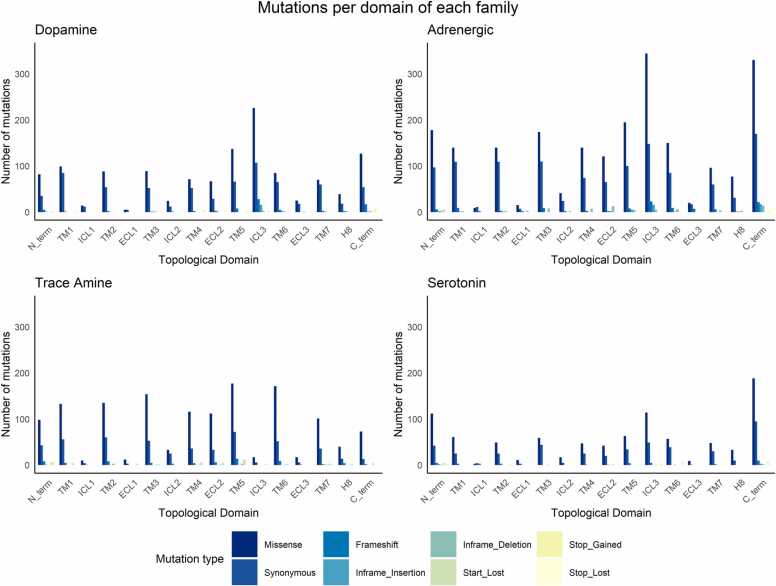
Distribution of the different mutations in topological domains of each family (dopamine, serotonin, adrenergic and trace amine receptors). The number of variants per topological domain as defined by the GPCR subfamily A17 molecular architecture of N- and C-terminal regions, seven TM helices (TM1 to 7), three extracellular loops (ECL1 to 3) and three intracellular loops (ICL1 to 3).

An analysis of the distribution of mutations over the topological domains of all receptors in the database as well as for the receptors of each family was performed (Fig. 3). The highest number of mutations was found in the TM region, with a total of 4981 mutations, with missense being the most frequent (3045 mutations), followed by synonymous (1668 mutations), frameshift (129 mutations), stop-gain (91 mutations), in-frame deletion (33 mutations), in-frame insertion (14 mutations), and stop-loss (1 mutation). Mutations in these regions can promote several deleterious effects, since interactions between the helices contribute to building the functional tertiary structure of the GPCR, which plays a very important role in receptor folding and stability, ligand binding and ligand-induced conformational changes for G protein coupling [62]. Furthermore, it has been previously postulated that disease-causing nonsynonymous mutations of GPCRs occur more frequently within TMs than nondisease-causing nonsynonymous mutations [63]. For example, the Val194^5.40^Gly mutation found in our data has been shown to decrease the agonist binding affinity to the D_4_R receptor [64], [65]. The Thr164^4.56^Ile mutation found in TM4 of the β_2_-adrenoceptor receptor is also associated with receptor desensitization and a decrease in agonist binding affinity [66], [67]. Furthermore, in the TMs, we counted a total of 916 mutations in TM5, the TM with the highest number of mutations, followed by TM3 (768 mutations), TM6 (750 mutations), TM1 (737 mutations), TM2 (685 mutations), TM4 (594 mutations) and TM7 (531 mutations).

The TM region was followed by the ICLs (1393 mutations), C-term (1185 mutations), N-term (757 mutations), ECLs (724 mutations) and finally HX8 (280 mutations). Regarding ICLs and ECLs, ICL3 was the one most enriched in known mutations (1128 mutations), followed by ECL2 (528 mutations). ICL regions are important for receptor interactions with signaling and regulatory proteins [63].

GPCR crystal structures have shown structural conservation in ICL1 and high levels of variability in ICL2 and ICL3, suggesting dynamic and/or unstable conformations of the last two regions [68]. This is in line with our data, which showed that ICL1 was the intracellular domain that mutated the least, and ICL3 the most. Moreover, ECLs were highly diverse with respect to both sequence and length, even when comparing subtypes of the same receptor family. ECL2 is known to vary in length between GPCR classes, resulting in distinct conformations, while ECL1 and ECL3 are short and often have disordered structures [69]. Although all ECLs have their own functions, ECL2 is the domain that plays important roles, including the formation of a disulfide bond with TM3, the glycosylation of ECL2, and participation in receptor activation, ligand binding and allosteric ligand function [70]. Our data demonstrated that ECL2 was indeed the extracellular loop with a higher number of reported mutations, which can contribute to destabilizing its functions.

The C-term is the third most mutated region, and it is involved in GPCR signaling, namely, in interactions with proteins that mediate GPCR signaling [63]. For example, the His452Tyr mutation found in the C-terminus of 5-HT_2A_R was reported to blunt intracellular calcium mobilization, decrease the response to clozapine and alter the kinetics of receptor desensitization [71], [72], [73]. The C-term is followed by the N-term, which is involved in ligand binding, activation, and downregulation [63]. Gln27Glu mutation, another modification identified in the β_2_-adrenoceptor, is responsible for blunt agonist-promoted downregulation [74]. The Cys23Ser mutation of 5-HT_2 C_R was revealed to decrease agonist binding affinity [75].

This trend in the global distribution of mutations by topological domain was not followed completely when analyzing each family individually (Fig. 3). In the dopaminergic family, the highest number of mutations was found for ICL3 (383 mutations), followed by TM5 (214 mutations) and C-term (210 mutations). Moreover, TM5 and TM1 were more mutated than the other TMs. In the adrenoreceptor family, C-term accounted for the most mutations (573 mutations), followed by ICL3 (548 mutations), TM5 (321 mutations), TM3 (302 mutations), N-term (297 mutations), and TM1 (263 mutations). Of the three extracellular loops, ECL2 showed the highest number of mutations (204 mutations). In the serotonin family, the C-terminal domain showed the highest number of mutations (308 mutations). ICL3 and ECL2 were the loops with more nucleotide changes (172 and 65 mutations, respectively) compared to the other corresponding regions. Regarding the TM domains, it was in TM3 that the highest number of mutations was found (105 mutations). Finally, in the TAR family, the number of mutations in each transmembrane domain was more distributed. The domain with the highest number was TM5 (278 mutations), followed by TM6 (236 mutations), TM3 (215 mutations), TM2 (207 mutations), TM1 (198 mutations), TM4 (163 mutations) and TM7 (144 mutations). Unlike other families, ICL2 was the intracellular loop with the most mutations (61), and the N-term had more mutations than the C-term (164 and 94 mutations, respectively. Furthermore, the most mutated extracellular loop was ECL2 (157 mutations). For all families, the ICL1, ECL1 and ECL3 domains were the ones that mutated the least, suggesting that these domains are the most conserved ones. Since the domains have different lengths, we normalized the data using a 3-residues window (data not shown). This normalization did not affect the results as the impact for any given domain was identical.

In our subsequent analyses, we focused on missense mutations, as they promote changes in the amino acid sequence and, consequently, may be involved in loss or gain of function, structural alterations, localization, signaling and ligand binding [76], [77]. These modified receptors can promote different pathways that may be involved in disease development and altered responses to GPCR-targeting drugs [78].

### 3.3 Solvent accessible surface area and mutability

The solvent accessible surface area (SASA) of proteins is a key feature for determining protein folding and stability [79], [80]. SASA is also important for functional annotation of disease-related protein variants [81], [82]. Here, we used rASA to split the receptor residues within the surface (rASA>25%) and interior (rASA<= 25%) regions (see also the “Substructures” and “Structures Representations” sections in the MUG database website). A correlation between this feature and the number of missense mutations was made using a one-way ANOVA test (p < 0.05) (Table A.1).

On average, the number of mutations per residue in surface residues was higher than that in the interior region, with averages equal to 0.57 ± 0.78 and 0.49 ± 0.73 (p-value = 5.35e-07), respectively, which was statistically significant. This clearly suggests that surface residues, which are more exposed to solvent, are more susceptible to having more mutations per residue. This evidence can be observed in all receptors, especially in D_1_R, D_2_R, D_3_R, 5-HT_2A_R, 5-HT_2 C_R, α_1B_-adrenoceptor and α_2 C_-adrenoceptor, for which the difference was statistically significant, while this was not the case for the other members of subfamily A17.

Some studies revealed a relationship between solvent accessibility of a residue and site-specific rate variation, suggesting that buried sites are more conserved and evolve slower than exposed sites [83], [84], [85], [86], [87], [88], [89], [90], [91], which is in line with our findings. In addition, it is well established that mutations in interior residues are more pathologic than in residues more exposed to the solvent [92], [93], [94].

### 3.4 Missense mutation distribution over the BW positions and relation with relevant residues

**Fig. 4:**
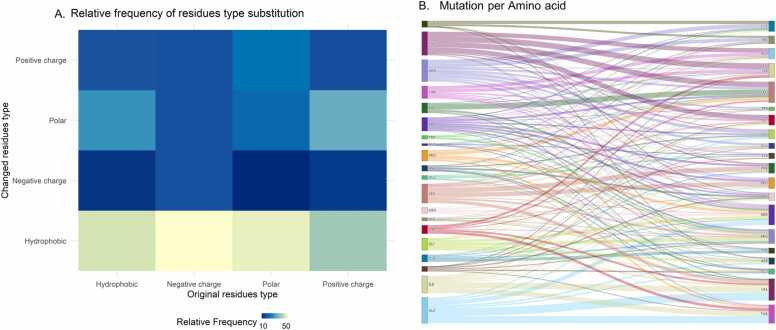
Missense mutations for all 25 receptors of GPCR subfamily A17 in the database. (**A**) Missense mutations per group of amino acids (hydrophobic, polar, negative charge and positive charge) and (**B**) per amino acid.

Within the GPCR subfamily A17, mutations preferentially lead to the occurrence of new hydrophobic residues at key points (Fig. 4A). However, the hydrophobic insertion and packing of the TM helices are dependent on the cumulative properties of the entire TM segment. Thus, a punctual alteration of hydrophobicity or van der Waals specificity at a site may not result in major structural and functional changes. Specifically, in TM regions, the loss of polar and ionizable residues leads to the loss of hydrogen bonds and strong electrostatic interactions, which play dominant roles in helix-helix interactions and, consequently, in protein folding within membrane domains [95].

As it can be analyzed in the MUG database website (“Overall” section), Ala residues were the ones found to mutate the most, preferably into Val and Thr (Fig. 4B). Val was the second most mutable amino acid, followed by Arg. In addition to the hydrophobic Ala and Val residues, Ser and Thr residues are often mutated, possibly since these polar residues do not play a significant stabilization role in promoting significant TM helix association [96], [97]. Furthermore, our data revealed that Ser and Thr in TMs mutate more often than the same residues outside of TMs, i.e., 51.4% and 59.7% of Ser and Thr mutations, respectively, were shown to occur in the TMs. According to data published in the MENSAdb [98], which contains features of dimer surfaces of membrane proteins and their interfacial regions, Ser and Thr are less conserved in the noninterfacial region, and the remaining polar and/or charged amino acids are more conserved in this region than in the regions of nonsurface residues and interfacial surface residues, which is in line with our findings. In contrast, polar and/or charged amino acids, such as Gln, Glu, Lys, Asp, His and Asn, play an important role in the stabilization of helical membrane proteins [96], [97], [99], [100], [101], suggesting that mutations in these residues are associated with protein malfunction [95]. In fact, these amino acids were rarely mutated, except for Asp and Asn, which preferentially mutated into Asn and Ser, respectively. Thus, the Asp residue completely changes the microenvironment charge character. Furthermore, Trp is one of the least mutated residues within this dataset, which could be related to its well-known function of supporting the self-assembly of TM helices [102]. Thus, mutations in hydrophobic Trp may reduce the self-interaction of the transmembrane segments without affecting their efficiency of membrane integration [102].

Analysis of the missense mutations for each residue of the transmembrane domains (TM 1–7), using the BW nomenclature, revealed that there were some residues that stood out for having a high number of mutations, such as Lys/Arg/Asn^6.29^ (29 mutations), His/Gln/Tyr/Gly/Leu/Lys/Ala/Val/Thr/Arg^1.32^ (27 mutations) and Arg^3.50^ (24 mutations), and others that stood out for not having any mutation Gly/Pro^1.24^, Leu/Arg/Gly/Tyr^1.27^, Thr^4.34^, Tyr^4.36^, His/Arg^4.66^, Lys/Gln^5.83^ and Asn/Ser^5.84^. We observed for most of the receptors that Arg^3.50^ undergoes the most mutations, followed by Pro^7.50^ (17 mutations) and Asp^2.50^ (15 mutations). As shown in Fig. A.1, when looking at each family individually, there is some diversity in the number of mutations per residue. In the serotonin family, no mutation was found at conserved residue Pro^7.50^, despite being one of the most mutated residues in the entire dataset. In addition to this finding, no mutations were found for the conserved residues Trp^4.50^ and Pro^6.50^. In the adrenergic family, the Lys/Arg^6.29^ residue was found to be highly mutated (16 mutations) in comparison with other families (dopamine - 4 mutations, serotonin - 3 mutations, trace amine - 6 mutations).

**Fig. 5:**
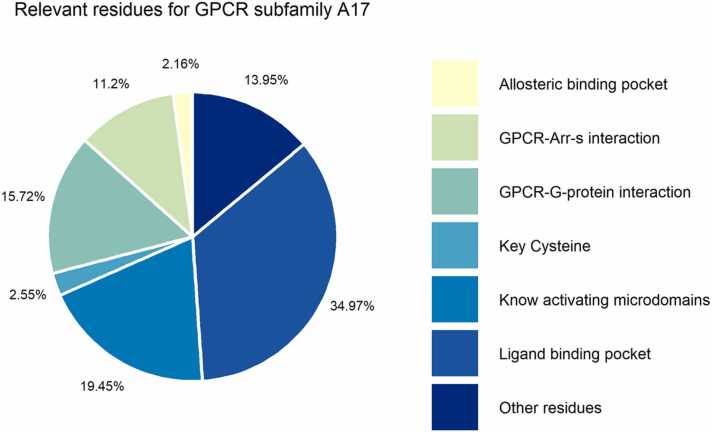
Pie chart showing the proportion of mutations found for each relevant area for all the members of subfamily A17.

Missense mutations can affect GPCR basal activity, ligand binding, interactions with G proteins and β-arrestins and cell expression [19]. The basal activity of GPCRs is defined by intramolecular constraints, which limits the flexibility of GPCRs and their ability to adopt a certain conformation in which they activate the G protein in the absence of a ligand. Consequently, mutations in activation-relevant microdomains can affect GPCR basal activity. Mutations in ligand binding pockets can affect agonist affinity, efficacy, or receptor selectivity. Such residues involved in ligand binding are mainly found in transmembrane domains and ECLs [103]. Mutations in the allosteric binding pocket can also affect ligand binding to the orthosteric pocket, which was reported to result in activation of more than one G protein or β-Arrestin [12], [104]. Furthermore, mutations of residues involved in GPCR-β-Arrestin interaction could possibly inhibit the binding of β-Arrestin and subsequent internalization of the receptor alternative independent signaling pathways. Additionally, mutations in the C-terminal and key cysteine residues have been reported to promote receptor instability and malfunction [103]. Consequently, all mutations in all these regions may alter or inhibit downstream signaling pathways and physiological responses of the receptor. Hence, we mapped the mutations according to regions relevant for activating the receptor for subfamily A17 into the following categories: ligand binding pocket, allosteric binding pocket, known activating microdomains, key cysteine residues, GPCR-G protein interaction, GPCR-Arr interaction, and other relevant residues, to state that there were certain cohorts of mutations affecting a specific function of the receptors (Fig. 5). In the MUG database website, we provide all the GPCR structures under study with these respective identified categories in the subsection "Relevant residues" of the "Structures Representations". Thus, it is possible to have a structural perception of the selected categories. We also suggest analyzing the mutations in the GPCR structures in the section "Structures Mutations".

### 3.5 Overview of mutations in specific groups of relevant residues

The highest number of mutations was found for residues that belong to the ligand binding site (35%), followed by residues that belong to known activating microdomains (19%) and residues that participate in GPCR-G protein interactions (16%). The fewest mutations accounted for regions comprising key cysteines and allosteric binding pockets (Fig. 5).

Mutations in residues belonging to the ligand binding site of the receptors can change the affinity at which a response is achieved (decreased for GOF mutations and increased for LOF mutations); the efficacy of a ligand, which can be increased with a mutation that facilitates the formation of the conformation of the active receptor, which provides a more favorable interface for the activation of the G protein and receptor selectivity [19]. The mechanisms that alter specificity appear to be different when the mutations are either in the N-terminal domain or in the TM domains. Mutations in the N-terminal domain of GPCRs can change the recognition specificity and accessibility of the receptor, while mutations in TMs may change the energy barrier for activation by an alternative ligand, thereby altering the functional selectivity of the receptor [19], [103], [105], [106]. We found a high number of mutations at positions Phe/Cys/Tyr^6.51^ (16 mutations), Asp^3.32^ (11 mutations), Ser/Ala/Leu^5.46^ (8 mutations), Lys/Val/Phe/Ser/Ala/Gly^2.61^ (7 mutations) and Lys/Ser/Thr/Ile/His/Arg^2.64^ (7 mutations). The Phe^6.51^ residue was found to mostly change into Leu, losing the aromatic ring but remaining in the hydrophobic group. Likewise, Phe^6.51^ was also mutated into Try and Ile (both hydrophobic) and Cys. The Asp^3.32^ residue mutated mostly to Glu (does not change the charge), followed by His (changing to positive charge), Asn and Gly (losing charge). Although mutations in ligand binding site were found in all families, it was in adrenergic family, essentially in α_1A_-adrenoreceptor, α_1D_-adrenoreceptor, and α_2A_-adrenoreceptor, that a high number of these residues showed to mutate the most.

The allosteric binding site is an alternative binding site distinct from the orthosteric site, and the binding of allosteric modulators potentiates or inhibits activation of the receptor by its natural ligand [107]. Allosteric ligands and their binding to allosteric binding sites influence the ability to fine-tune the response to an orthosteric ligand in a time- and spatially dependent manner and may confer signaling bias and probe dependence, further contributing to the possibility for remarkably precise pharmacological modulation [108], [109], [110], [111]. Our analysis showed only a few mutations in allosteric binding sites. Residues with a high number of mutations were found for positions Arg/Lys^34.52^ (ICL2, 4 mutations) and Ser^8.47^ (HX8, 2 mutations). The Arg/Lys^34.52^ residue was found to be mostly mutated into Gln and Trp and Glu and Met, respectively, with equal occurrence. The change to residues with other physicochemical properties may have significant effects. Ser^8.47^ (HX8) was mutated to Ile and Asn. Only in D_1_R and β_2_-adrenoreceptor were found mutations in residues belonging to allosteric binding site.

Upon GPCR activation, the cytoplasmic ends of TM4 and TM5 were reported to move, forming an interface for G protein binding and activation [19], [112]. Mutations in residues belonging to the GPCR-G protein interface can interfere with the process of coupling to downstream effectors by changing the exposure or the structure of the interaction interface [19], [113]. In subfamily A17, the residues Arg/Lys/Asn^6.29^ (8 mutations), Arg/Lys^6.24^ (6 mutations) and Ile/Ala^8.48^ (5 mutations) stood out in the number of known mutations. Arg^6.29^ mutated similarly to Pro, Gln, Trp, Met, Leu, His and Cys, and Lys^6.29^ mutated into Thr. Except for the Arg^6.29^His mutation, all other mutations did not significantly change the physicochemical properties of the residue present in the position. Likewise, this was observed for the residue at position 6.24. Furthermore, the Ile/Ala^8.48^ residue mutated into Thr and Asn and to Thr and Val, respectively. Therefore, there was a switch from hydrophobic to polar amino acids. Mutations in residues that interact with G protein were found essentially in dopamine (D_1_R, D_2_R, D_3_R, D_4_R, D_5_R) and adrenergic (α_1B_-adrenoreceptor, α_2B_-adrenoreceptor, and β_1/2/3_-adrenoreceptors) families. Many GPCRs can interact with a different downstream effector, β-arrestin [114], [115], [116]. However, there is weak information reported on the possible effects of mutations at the site of interaction of GPCRs with β-Arrestin. In this dataset, Arg^34.55^, Ile/Val/Gly^5.64^ and Arg/Lys^6.29^ were the ones that underwent the most mutations. Arg^34.55^ mutated to Gly (2 mutations) and to Gln, Leu and Cys. Ile/Val/Gly^5.64^ mutated to Leu, Ala, Met, Cys and Ser (1 mutation each). Arg/Lys^6.29^ mutated to Pro, Gln, Trp and Thr (1 mutation each). Dopamine family receptors are the ones with higher number of mutations in residues that interact with arrestin.

Microdomains determine the level of basal activity, which limits the flexibility of GPCRs and the ability of receptors to adopt conformations that can activate the G protein, even without agonist binding. These microdomains are the DRY motif, PIF motif, CWxP motif and NPxxY motif [117], [118]. They play an important role in mechanism of activation, because they make different interactions in the active and inactive state of the receptor [117]. Thus, mutations in these motifs may break some import interactions established to stabilize the receptor. Mutations in these motifs were also identified and analyzed. An important motif is DRY, with the conserved Arg at position 3.50. This motif is located at the boundary between TM3 and ICL2 and is directly involved in the regulation of receptor conformational states and/or in the mediation of G protein activation of class A GPCRs [113], [119], [120]. Furthermore, Arg^3.50^ is considered a key residue in GPCR signal transduction since replacement of Arg^3.50^ with different amino acids may modify the transduction capacity of the receptor [119], [121]. Mutations in Arg^3.50^ can generate two different phenotypes in GPCRs: increased agonist-independent or constitutive receptor activity [120], [122], [123], [124]. However, the opposite may happen, and mutations in Arg^3.50^ do not necessarily lead to increased constitutive activity but can still affect receptor folding [125], [126]. Among the subfamily A17, D_5_R, 5-HT_2A_R, 5-HT_2B_R, α_1A_-adrenoceptor, α_1B_-adrenoceptor, α_2B_-adrenoceptor, α_2 C_-adrenoceptor, β_3_-adrenoceptor, TA_1_R, TA_5_R, and TA_9_R accounted for the most mutations at position 3.50. The most frequent substitutions of Arg^3.50^ involved Cys (10 mutations), Ser (5 mutations), and His (5 mutations) but also involved Leu (3 mutations), Gly (3 mutations), Pro (2 mutations), Thr (2 mutations) and Lys (1 mutation). The high number of substitutions in this residue suggests that Arg^3.50^ is very sensitive to sequence variations, and they may be linked to pathological outcomes in several GPCRs [120], [127], [128], [129]. Mutations in 3.49 were found in D_2_R, α_1A_-adrenoceptor, α_1D_-adrenoceptor, β_1_-adrenoceptor, β_3_-adrenoceptor, TA_1_R, TA_2_R, TA_5_R, TA_6_R, TA_8_R, and TA_9_R. Asp^3.49^ mutated mostly to Glu (5 mutations), Asn (4 mutations), Gly (4 mutations), and Tyr (3 mutations). Tyr^3.51^ mutates to Cys (3 mutations) and His (2 mutations) and these mutations were found in D_3_R, α_1A_-adrenoceptor, α_1D_-adrenoceptor, α_2B_-adrenoceptor, TA_8_R, and TA_9_R.

Arg^3.50^ is also known to form an ionic lock in class A GPCRs, which is a salt bridge between two highly conserved amino acids at the bottom of TM3 (Arg^3.50^) and TM6 (Asp/Glu^6.30^), which has been associated with modulation of basal activity [130], [131]. This interaction constrains the receptor to an inactive state by keeping the cytoplasmic ends of TM3 and TM6 in proximity [130]. Mutations of Asp^6.30^ to different amino acids break this salt bridge, relieving the constraint and thereby increasing constitutive activity in several GPCRs [132], [133], [134], [135]. Most of the receptors in subfamily A17 had the Glu residue at this position, and mutations of this residue were found in the receptors D_2_R, 5-HT_2B_R, α_1A_-adrenoceptor, α_2A_-adrenoceptor, β_1_-adrenoceptor, β_3_-adrenoceptor, TA_5_R, TA_6_R, TA_8_R and TA_9_R. Glu^6.30^ mutated mostly to Asp and Lys. When it mutated to Lys, there was a change in charge from negative to positive, which highly increased the likelihood of the salt bridge breaking.

The PIF motif is constituted by Pro^5.50^, Ile^3.40^ and Phe^6.44^ conserved residues and forms an interface between TM5, TM3 and TM6 [136], and the PIF motif is only conserved in a few GPCRs, such as the β_2_-adrenoceptor and 5-HT family [104], [112], [136]. Upon receptor activation, the subtle agonist-induced changes in the ligand-binding site cause repacking of side chains of these residues near the ligand binding site [103], [137]. As a result, conformational changes in the transmembrane core are induced, such as a rearrangement at the TM3–TM5 interface and the formation of new noncovalent contacts at the TM5–TM6 interface [103]. In fact, these residues were mutated in 5-HT_2B_R, 5-HT_6_R, α_1A_-adrenoceptor, α_1D_-adrenoceptor, α_2A_-adrenoceptor, and β_3_-adrenoceptor receptors. Ile^3.40^ mutated to Asn, Thr, and Val, Pro^5.50^ mutated to Ser and Thr, and Phe^6.44^ mutated to Leu. The mutations in Pro^5.50^ changed the polarity of nonpolar to polar and may result in the formation of noncovalent contacts at the TM5–TM6 interface.

The CWxP motif of TM6 is highly conserved in class A GPCRs and is constituted by Cys^6.47^, Trp^6.48^ and Pro^6.50^. This motif is the basis of the rotamer toggle switch hypothesis and plays a role in active forms of GPCRs [138]. The Cys^6.47^ residue interacts with Asn^7.49^ in the inactive state and forms a gap between Asn^7.49^ and Asp^2.50^. After activation, this interaction is disrupted, and Asn^7.49^ interacts with Asp^2.50^ [139]. In inactive state, Trp^6.48^ interacts with a structural water molecule of the hydrogen-bond network which stabilize this conformation, whereas in active state the Trp^6.48^ side chain form an aromatic interaction with the highly conserved Phe^5.47^ of TM5 [117], [140]. Pro^6.50^ creates a kink in TM6 and works as a pivot for helical movement during receptor activation [117], [140], [141]. All these interactions can be disrupted when these residues are mutated. Trp^6.48^, which is very conserved among class A GPCRs, was found to mutate very often, especially in D_4_R, 5-HT_6_R, α_1A_-adrenoceptor, TA_1_R and TA_5_R receptors. Trp^6.48^ was shown to preferably mutate into Cys (8 mutations) but also into Gly (2 mutations), Arg (2 mutations) and Leu (2 mutations). The neighboring residue Cys^6.47^ was found to be mutated only in D_4_R, α_2A_-adrenoceptor, TA_1_R and TA_2_R and only once into Trp, Ser, Gly and Arg. Pro^6.50^ mutated only in D_4_R, α_1B_-adrenoceptor, β_3_-adrenoceptor, TA_1_R and TA_8_R and mutated more to Leu (4 mutations) but also to Ala (2 mutations) and Ser (1 mutation).

The NPxxY motif also belongs to microswitches and may contribute to the internalization of receptors and is involved in the transition from the ground state to active forms of GPCRs. The NPxxY motif is composed of three conserved residues: Asn^7.49^, Pro^7.50^ and Tyr^7.53^. The Asn^7.49^ residue is essential in the stabilization of both the inactive and active states of GPCRs and in the regulation of the conformational transition of GPCRs. Thus, replacement of Asn^7.49^ for other residues may modify the TM7 conformation and produce a change in signalization patterns [142]. The conserved Pro^7.50^ residue also acts as a rotamer toggle switch [117]. Tyr^7.53^ plays a role in receptor activation in all class A GPCRs [143]. In the inactive state of the receptor, Tyr^7.53^ was reported to form contacts with residues Phe/Tyr^8.50^ and Val/Leu/Met^1.53^. Upon receptor activation, Tyr^7.53^ forms a new contact with residue 3.46 [143]. Therefore, mutations in the Tyr^7.53^ residue may reduce G protein activation [143], [144]. The receptors D_4_R, 5-HT_2B_R, 5-HT_2 C_R, α_1B_-adrenoceptor, α_1D_-adrenoceptor, α_2 C_-adrenoceptor, β_1_-adrenoceptor, TA_1_R, TA_5_R, TA_8_R and TA_9_R showed mutations at Asn-7.49 into different residues, including Ser, Lys, and Asp. In contrast, receptors D_5_R, α_1A_-adrenoceptor, α_1D_-adrenoceptor, β_3_-adrenoceptor, TA_2_R, TA_8_R and TA_9_R were found to mutate frequently at Tyr^7.53^ and preferably into Lys and Cys.

Asp^2.50^ is a conserved residue on TM2 that is known to form key interactions with sodium [145]. The high conservation of Asp^2.50^ among GPCRs suggests its structural importance for GPCR function. The carboxylic group with negatively charged Asp^2.50^ interacts by electrostatic interaction with the positively charged sodium ion. Therefore, the replacement of Asp by nonnegatively charged residues generates insensitivity to sodium [146], [147]. D_4_R, 5-HT_2B_R, α_1A_-adrenoceptor, α_1B_-adrenoceptor, α_1D_-adrenoceptor, α_2B_-adrenoceptor, β_3_-adrenoceptor, TA_2_R, TA_5_R, TA_6_R and TA_9_R were the receptors that were shown to have mutations at this position. Mutation of Asp^2.50^ mostly led to changes into Asn (polar) and Gly (nonpolar).

The conserved Cys^45.50^ (ECL2) was reported to form a disulfide bridge between ECL2 and the top of TM3 (Cys^3.25^) [148]. Since disruption of the conserved TM3-ECL2 disulfide bond was reported to be unfavorable for many GPCR A families, mutations in Cys^45.50^ are associated with a loss of function in GPCRs [70]. Mutations of Cys^45.50^ were found for α_2B_-adrenoceptor, TA_1_R, TA_6_R, TA_8_R and TA_9_R and preferably mutated into Phe (4 mutations), followed by Arg (2 mutations), Tyr (2 mutations), Ser (1 mutation), and Gly (1 mutation).

### 3.6 Relevant residues and pathogenicity of missense mutations

A lot of diseases are associated with members of subfamily A17, such as neurodegenerative diseases (Alzheimer’s disease and Parkinson’s disease), schizophrenia, hypertension, obesity, addiction, major depression, attention deficit hyperactivity disorder, fibromyalgia, and diabetes mellitus type 2 [149], [150]. Therefore, since mutations may be associated with neurodegenerative disease, it is relevant to study the pathogenicity of each mutation.

12 of 596 missense mutations found in relevant residues for GPCR subfamily A17 are homozygous mutations, which means that those individuals carry two copies of the mutation. Homozygous mutations were found 8 genes of GPCR subfamily A17: D_1_R (Ser259Tyr), D_3_R (Val136^34.51^Ile, Arg323^6.29^Gln), D_5_R (Met75^2.38^Thr), β_2_-adrenoceptor (Asn69^2.40^Ser), TA_1_R (Asn300^7.49^Lys, Ile104^3.33^Val), TA_5_R (Asp114^3.32^Val), TA_6_R (Cys291^7.33^Tyr, Asp281^6.58^Ala, Thr93^2.65^Ala), and TA_8_R (Asp276^6.54^Ala). However, until now, only the missense mutation Val136^34.51^Ile in DRD_3_ gene is known to express a clinical phenotype of hereditary essential tremor 1 and is likely benign [151].

Given the importance of homozygous mutations, we predicted their functional effect, using available pathogenicity prediction tools (Table A.2). It was already expected different results for each tool as their overall performance is lower than 90%. Even so, the FATHMM, PROVEAN, MutaFrame, SNPs&GO and SuSPect tools classified most mutations as benign, and on the contrary the remaining programs classified most mutations as pathogenic. Of the 12 homozygous mutations under study, we found that 4 were classified by most programs as pathogenic (Table A.2). The mutations Ser259Tyr (D_1_R) was classified as pathogenic in 5 of the 9 tools used in this analysis, Asp114^3.32^Val (TA_5_R) in 6 of the 9 tools, and Asn69^2.40^Ser (β_2_-adrenoceptor) and Asn300^7.49^Lys (TA_1_R) in 7 of the 9 tools. These 4 homologous mutations classified as pathogenic exhibited allele frequencies in the range of 1e-03. Moreover, they have not yet been reported in the literature associated with any disease, and their role in diseases related to the respective receptors should be assessed.

**Fig. 6:**
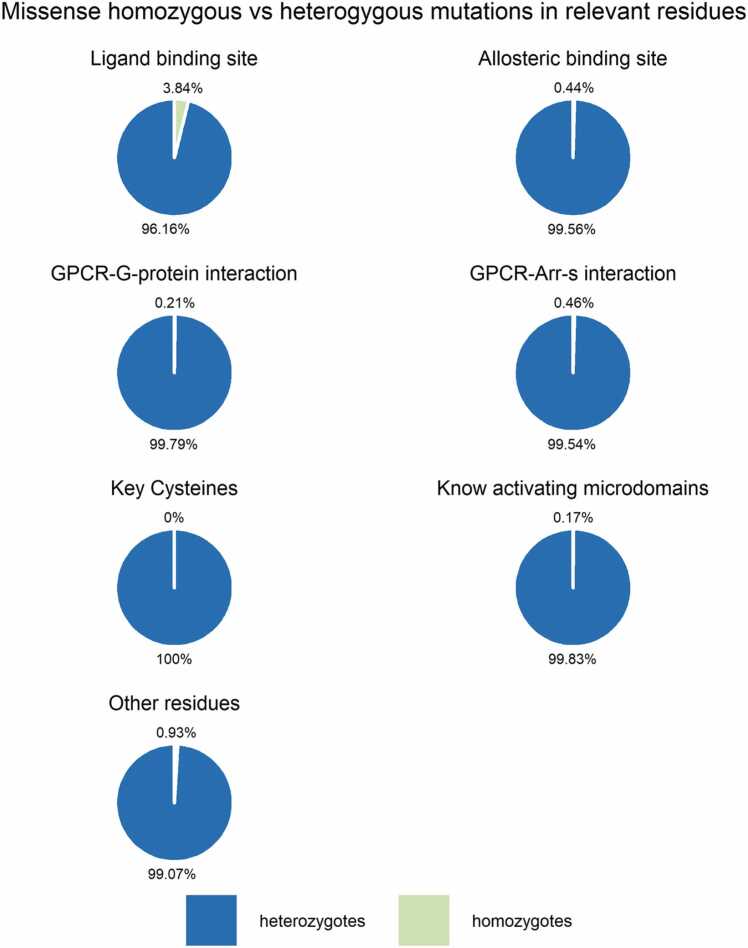
Percentage of homozygous vs heterozygous missense mutations in each relevant residues group. Groups of relevant residues include ligand binding site, allosteric binding site, G protein binding site, arrestin binding site, microdomains, cysteines, and other residues.

Our homozygous vs heterozygous missense mutation analysis in relevant residues verified a higher frequency of heterozygous mutations in all groups (Fig. 6). Besides that, homozygous mutations were found with higher percentage in ligand binding sites (3.64%) of our receptors, followed by other residues (0.93%), GPCR-arrestin interaction (0.48%) and allosteric binding site (0.44%). In key cysteines no homozygous mutation was found.

The remaining mutations were identified in heterozygote individuals. Of the 596 relevant missense mutations, 364 were classified as pathogenic and 232 were classified as benign based on analyses by 9 pathogenicity predictors.

In dopamine family, the D_1_R receptor, Ser259Tyr mutation (G protein and β-Arrestin interaction site) was highly expressed. This mutation is one of the homozygous mutations and was classified as pathogenic. In fact, Ser259 residue is one of the C-term serine residues where GRK phosphorylation occurs, therefore the mutation from small size and polar Ser to large size and aromatic Tyr suggests decreasing phosphorylation [152]. Ile125^3.54^Val and Val200^5.44^Ala (G protein and β-Arrestin interaction site) were classified as benign. Of 19 missense mutations of D_1_R, 12 were classified as pathogenic, for example Phe288^6.51^Leu and Phe313^7.35^Leu in ligand binding site, and Leu143^4.45^Pro in allosteric binding site, which exhibited allele frequencies in the range of 1e-06. So far, none of them have been associated with a known disease. In the D_2_R receptor, 14 of 23 mutations were considered pathogenic and had allele frequency around 1e-06. For β-Arrestin interaction site Val215^5.64^Leu was benign, Arg219^5.68^Cys was pathogenic, Lys226^5.75^Arg was benign and Glu368^6.30^Asp was pathogenic. Asp131^3.49^Asn and Glu368^6.60^Asp of microdomains were both classified as pathogenic. Among them Arg151^4.41^Trp (pathogenic) and Val111^3.29^Ile (benign) were found in G protein interaction site. Regarding D_3_R, 11 of 38 missense mutations were considered pathogenic. Ala38^1.41^Thr (other residues), was the most frequent mutation, but was classified as benign. Val136^34.51^Ile (β-Arrestin interaction site) was also identified as homozygous mutation, classified as benign. Despite being considered benign, mutations Ala38^1.41^Thr and Val136^34.51^Ile are associated with hereditary essential tremor 1 disease [151], [153]. The Phe346^6.52^Leu mutation, involving a residue at the ligand binding site, was pathogenic. Besides the last one, Arg318^6.24^Trp (G protein interaction site), Pro135^34.50^His (β-Arrestin interaction site), Tyr129^3.51^Cys (know activating microdomains), and Try66^2.41^Cys (other residues) were classified as pathogenic and exhibited allele frequencies in the range of 1e-05. At the D_4_R receptor, the binding site residues were the ones found to be mutated the most, being Pro351Gln mutation predominant. For this receptor, 32 of 76 mutations were pathogenic, e.g., Thr67^2.37^Met (β-Arrestin interaction site), Asp115^3.32^His (ligand binding site), Phe201^5.47^Ser (other residues), Cys406^6.47^Trp and Trp407^6.48^Gly (know activating microdomains), which exhibited allele frequencies in the range of 1e-04. In the D_5_R receptor, Met75^2.38^Thr and Asn74^2.37^Lys mutations (β-Arrestin interaction site) were classified as benign. Residues that were reported to be relevant for the ligand binding site, such as Asn316^6.55^Ser which was classified as pathogenic. Moreover, 25 of 52 missense mutations of D_5_R were considered pathogenic and exhibited allele frequencies in the range between 1e-06 and 1e-05.

Regarding serotonin receptors, at the 5-HT_2A_R ligand binding site, the Ile152^3.29^Val mutation was the most frequent mutation (allele frequency of 3.19e-05). 12 missense mutations were classified as pathogenic in 5-HT_2A_R, including Ala321^6.33^Val and Arg173^3.50^Cys, with an allele frequency in range of 1e-06. In the 5-HT_2B_R, 15 of 25 mutations were considered pathogenic, such as Glu319^6.30^Lys (ligand binding site), Arg153^3.50^His (PIF motif), Ile143^3.40^Asn (PIF motif), and Thr140^3.37^Ile (G protein binding site) with allele frequencies in the range between 1e-06 and 1e-05. For the 5-HT_2 C_R receptor, only 3 missense mutations were classified as pathogenic, including Ala222^5.46^Asp (ligand binding site) and Asn364^7.49^Ser (NPxxY motif), but also Phe214^5.38^Val (ligand binding site), which exhibited allele frequencies in the range of 1e-06. In the 5-HT_6_R, only 2 of 20 missense mutations were classified as benign with exhibited allele frequencies in the range of 1e-06. In the ligand binding site, the Ala192^5.42^Thr was found to be the most frequent mutation, followed by Cys110^3.36^Trp and Phe284^6.51^Ser. In the microdomains, the mutations Ile114^3.40^Asn, Pro200^5.50^Ser, Tyr320^7.53^His and Trp281^6.48^Cys stood out. With exception of Ala192^5.42^Thr, all the other mentioned mutations were classified as pathogenic.

26 of 40 missense mutations in α_1A_-adrenoceptor receptor were classified as pathogenic. In the α_1A_-adrenoceptor receptor, Arg166Lys (ligand binding site) was one of the most frequent mutations and was considered benign. The Pro293^6.56^Ser and Tyr194^5.48^Ser (ligand binding site) were the second most frequent mutations and were both classified as pathogenic. Besides that, Arg342Cys (other residues) was also considered pathogenic and exhibited allele frequency in the range of 1e-04. For the α_1B_-adrenoceptor receptor, the Phe303^6.44^Leu mutation located in the PIF microdomain was the most highly expressed. As with the Asn344^7.49^Asp mutation found in the NPxxY motif. Asp125^3.32^Ala and Asn190Ser were highly found in ligand binding site. Only 3 mutations were classified as benign, and all those mentioned above mutations were classified as pathogenic. In the α_1D_-adrenoceptor receptor, 16 of 22 missense mutations were considered pathogenic with allele frequencies in range of 1e-06. The Ala255^5.39^Thr (ligand binding site) was considered benign. Unlike the Pro241Leu and Pro241Arg (ligand binding site) were considered benign. In the NPxxY microdomain, the mutations Pro399^7.50^Arg and Asn398^7.49^Lys stand out and were both considered pathogenic.

For the α_2A_-adrenoceptor receptor, the Thr412^6.58^Met mutation was clearly shown to have the greatest impact, as it belongs to the ligand binding site and was highly expressed but was classified as benign. In addition, Glu204^45.51^Gln (benign), which was also part of the ligand binding site, was also found with some frequency. The Ile136^3.40^Thr mutation was the most evident and was in the PIF microdomain. The last mutation was classified as pathogenic as well as 9 other mutations from a total of 19. For the α_2B_-adrenoceptor overall, many mutations were found at the site of interaction with the G protein, including Arg44^12.51^Cys, Leu118^34.51^Val, Ser122^34.55^Pro and Leu118^34.51^Pro. Of 27 missense mutations, 21 were classified pathogenic with allele frequencies in the range between 1e-06 and 1e-05. In the α_2B_-adrenoceptor ligand binding site, the Cys96^3.36^Ser mutation stood out for being the most frequent and was considered pathogenic. For α_2 C_-adrenoceptor, 10 of 17 mutations were pathogenic with allele frequencies in the range of 1e-06. At the α_2 C_-adrenoceptor ligand binding site, the Ile182^4.56^Thr (pathogenic), Phe220^5.48^Leu (pathogenic), Ser213^5.41^Tyr (benign) and Val104^2.57^Leu (benign) stood out. For the microdomain group of α_2 C_-adrenoceptor, only one mutation in the NPxxY motif was found, Asn433^7.49^Ile (pathogenic).

For the β_1_-adrenoceptor receptor, the most prominent mutation was Asp356^7.32^His, which led to a charge exchange but was considered benign and was located at the ligand binding site. In the microdomain group, the Asp155^3.49^Gly mutation (pathogenic) was found in the DRY motif, the Glu319^6.30^Asp mutation (pathogenic) belongs to the ionic lock, and Asn373^7.49^Asp (pathogenic) belongs to the NPxxY motif. Val230^5.44^Ala and Asp155^3.49^Gly mutations were highly found in β_1_-adrenoceptor binding site to the G protein and β-arrestin, respectively, and were both considered pathogenic. For the β_2_-adrenoceptor receptor, the most evident mutation was Asn69^2.40^Ser, which belongs to the allosteric binding site. Furthermore, Asn69^2.40^Ser was also homozygous mutation (pathogenic) and was found to have detrimental effects on G-protein coupling [154]. In the β_2_-adrenoceptor ligand binding site, Asn301Ser and Phe193^45.52^Leu mutations were the most relevant. With exception of Asn301Ser, the other mutations were classified as pathogenic as well as 4 other relevant mutations of β_2_-adrenoceptor receptor, which exhibited allele frequencies in the range between 1e-06 and 1e-05. For the β_3_-adrenoceptor receptor, the Ser169^4.57^Leu mutation (ligand binding site), Pro343^7.50^Leu (NPxxY motif) and Glu287^6.30^Asp (ionic lock) stood out for their high frequency. Since only 2 of 23 β_3_-adrenoceptor relevant mutations were classified as benign, the mutations mentioned above were classified as pathogenic and most had an allele frequency around 1e-06.

Regarding trace amine receptors, in TA_1_R, 22 relevant missense mutations were identified: 15 classified as pathogenic and 7 as benign. For TA_1_R, the most frequent mutation was Asn300^7.49^Lys (pathogenic), which was found in the NPxxY motif. At the ligand binding site, the Ile104^3.33^Val mutation (benign) also stood out. In addition, Ile104^3.33^ residue was found to form hydrophobic interactions with ulotaront (TA_1_R agonist) in ligand binding site [155], suggesting that Ile104^3.33^Val mutation (also homozygous mutation) may influence the interaction with the ligand. For the TA_2_R receptor, the Try315^7.53^Cys mutation in the NPxxY motif was the most frequent (allele frequency of 2.66e-04), followed by the Trp302^7.40^Arg mutation (ligand binding site). These two mutations were considered pathogenic, as well as 15 other mutations in a total of 21 relevant mutations identified. Those heterozygous pathogenic mutations presented allele frequencies between 1e-04 and 1e-06. For the TA_5_R, only 1 mutation, Leu207^5.46^Ser, was classified as benign in a total of 22 relevant missense mutations identified. The Asp114^3.32^Val mutation appears to have an impact by losing the negative charge and switching to a hydrophobic residue. Indeed, it was classified as pathogenic. The mutation Arg132^3.50^Cys, also pathogenic and found in the PIF motif, stood out. In the TA_6_R, The Cys291^7.33^Tyr mutation, found in the ligand binding site, was the most frequent in the entire dataset. However, it was classified as benign. Other mutations, such as Thr93^2.65^Ala (benign) and Trp98^23.50^Arg (pathogenic), which are part of the ligand binding site, were also highly expressed. The majority of 23 mutations identified in TA_6_R were considered pathogenic (12 of 23 mutations) and exhibited allele frequencies in the range of 1e-06, with exception of Trp98^23.50^Arg which showed allele frequency in the range of 1e-04. For the TA_8_R receptor, the Asp276^6.54^Ala mutation (ligand binding site) was the most frequent but was classified as benign. Glu252^6.30^, which is part of the ionic lock, was mutated to Lys, changing the charge of the residue, and Pro272^6.50^, part of the CWxP motif, was mutated to Leu. Both mutations were classified as pathogenic. Likewise, this was also the case for a conserved Cys, Cys104^3.25^, which was mutated to Tyr. With exception of Asp276^6.54^Ala mutation and other 3 mutations, the other 13 mutations were considered pathogenic with allele frequencies in the range between 1e-06 and 1e-05. For the TA_9_R receptor, all mutations were classified as pathogenic. The Try311^7.53^Cys mutation found in the NPxxY motif stood out with an allele frequency of 2.49e-04.

In summary, all mutations identified in the key cysteines were found to be pathogenic, as well as most mutations identified in the receptor microdomains. This is in fact relevant, as these domains are fundamental for the stability, dynamics, and function of the receptors and, therefore, any anomaly can damage the conformation and function of the receptor. At the ligand binding site, of the 229 mutations identified, 133 of them are pathogenic. On the other hand, at allosteric binding pocket, GPCR-Arrestin interaction and GPCR-G protein interaction more benign than pathogenic mutations were identified.

### 3.7 Interactive application to explore GPCR subfamily A17 mutations

In this project, an interactive open access platform was developed to explore mutations in the subfamily A17 GPCRs. MUG provides a repository of natural mutations in subfamily A17 GPCRs and several interactive tools for data selection and analysis. This database contains information about individual receptors of each family, including original sequence, BW positions, amino acid changes and respective mutation type, topological location in the receptor structure, predicted orientation in membrane, SASA, rASA, interface region and the relevance of each residue. The MUG database is divided into 5 subsets, including MUG, Overall, Substructures, Structures Representations and Structures Mutations. A graphical panel interface is provided, allowing us to interactively display selected content according to receptor types, mutation type, amino acid exchange, topological domain, BW position, SASA and rASA values and relevant residue groups. Users can also assess the various structures of the various receptors obtained from GPCRdb, homology modeling and AlphaFold. The receptor structures have the structure residues colored according to the SASA, rASA, interface region, relevant residues, and the different types of mutations. In this way, the database should be used for the functional evaluation of natural variations.

## 4 Conclusions

GPCRs are a hot topic of pharmaceutical research due to their involvement in a wide variety of human physiological processes, including immunological, metabolic, and reproductive disorders, cancer, and neurodegenerative diseases [17]. More than 35% of all FDA-approved drugs target GPCRs, making them the largest family of proteins targeted by biopharmaceutical drugs [156]. Despite the clinical importance of GPCRs, genotype-phenotype relationship studies of GPCR natural variants have been scarce, and only a fraction of disease-associated GPCR mutations have been functionally characterized. Given the pharmacological and pathological importance of GPCRs, it is necessary to understand the relationship between genotype and phenotype, especially for the GPCR subfamily A17, whose available information is insufficient. For the first time, the distribution in a structural context and the possible impact of all known natural variants, mainly missense mutations, from all 25 receptors from GPCR subfamily A17 are provided.

A detailed characterization of the several mutations in this dataset of GPCR subfamily A17 provide insights as a first step for disease phenotype predictions. SASA values make it possible to identify which residues are most exposed and most susceptible to mutations. Identifying the location of the mutations, i.e., whether they belong to the receptor binding site, to the G protein or arrestin interaction site, to the allosteric binding site or to the characteristic microdomains of class A GPCRs, allows us to gain an insight of the effects they may have on the structure and function of the receptor, including the drug response, the signaling mechanism, the alteration of the native form of the receptor. Homozygous and heterozygous nature of mutations was also taken account, and respective pathogenicity.

Within the GPCR subfamily A17, residues mostly switch to hydrophobic residues, which may result in structural and functional changes, especially in the TM region. The current study demonstrated that the mutations are distributed throughout TM domains but are more prevalent in TM5 than in the other. We found a diversity of mutations in the diverse GPCR subfamily A17 structures, drawing attention to the considerable number of mutations in conserved residues, such as Asp^2.50^, Arg^3.50^ and Pro^7.50^, which can modify or disrupt the activation mechanism of GPCRs. Additionally, mutated residues were enriched at the ligand binding pocket, especially in the adrenergic family, affecting the affinity and efficacy of a ligand. Researchers that use the MUG database will also find mutations in allosteric binding sites (essentially in D_1_R and β_2_-adrenoreceptor), in key cysteines (especially in the trace amine family), and in receptor interaction sites with G-protein (especially in dopamine and adrenergic families) and with β-arrestin (essentially in the dopamine family). 11 of the 12 homozygous missense mutations are not yet clinically relevant and therefore should be explored. In addition, 364 heterozygous mutations identified in relevant groups of residues were considered pathogenic based on 9 pathogenicity prediction tools, being a starting point for their analysis: how they can change the structure and function of receptors and their association with diseases. They had also been identified in all key cysteines and virtually all residues that belong to microdomains, where any anomaly can cause receptor instability and lead to change function and disease onset.

From this analysis, an interactive open access platform was built, allowing an easier exploration of all retrieved analyses made on the mutational data. This information will increase as new pharmacological data become available. We believe that the platform and the respective data analysis framework will allow the entire community to have access to privileged information about non-synonymous natural variants of these receptors in the structural context, providing valuable insight for future research in the GPCR field. Furthermore, this detailed classification of all known natural mutations in GPCRs will help to understand deregulation and guide the appropriate therapy.

Although this project is dedicated to the A17 subfamily of GPCRs, it provides a starting point and a well-structured pipeline, and can be applied to other families and/or subfamilies of GPCRs.

## Funding

This work was supported by the 10.13039/501100008530European Regional Development Fund through the COMPETE 2020 - Operational Programme for Competitiveness and Internationalisation and Portuguese national funds via 10.13039/501100001871Fundação para a Ciência e a Tecnologia (FCT) [LA/P/0058/2020, PTDC/QUI-OUT/32243/2017, UIDB/04046/2020, DSAIPA/DS/0118/2020 and UIDP/04046/2020]. B.B., C.A.V.B and A.J.P. were supported by FCT through PhD-scholarships SFRH/BD/149709/2019, SFRH/BD/145457/2019 and SFRH/BD/144966/2019.

## CRediT authorship contribution statement

**Ana B. Caniceiro:** Data curation, Investigation, Formal analysis, Methodology, Software, Visualization, Writing – original draft. **Beatriz Bueschbell:** Writing – original draft & review, Methodology. **Carlos A. V. Barreto:** Methodology, Software. **António J. Preto:** Methodology, Software. **Irina S. Moreira:** Supervision, Conceptualization, Formal analysis, Funding acquisition, Resources, Writing – review & editing.

## Declarations of interest

The authors have no conflicts of interest to declare.
