# Supplementary material for: MUG: A mutation overview of GPCR subfamily A17 receptors
Source: Comput Struct Biotechnol J. 2022 Dec 21;21:586–600. doi: 10.1016/j.csbj.2022.12.031 (PMC9822836; doi:10.1016/j.csbj.2022.12.031)
Supplement: Supplementary file 1 — Supplementary material. [file mmc1.docx]

**Appendix A: Supplementary Data**

**MUG: a mutation overview of GPCR subfamily A17 receptors**

Ana B. Caniceiro, Beatriz Bueschbell, Carlos A.V. Barreto, António J. Preto and Irina S. Moreira

Corresponding author: Irina S. Moreira; E-mail: irina.moreira@cnc.uc.pt.

**Supplementary data 1: Solvent accessible surface area and mutability**

**Table A.1.** Correlation between surface/interior feature (rASA) and the number of missense mutations. Significance code indicates that (*) corresponds to statistically significant and () corresponds to statistically not significant.

| **Receptor** | **Significance** | **Number of missense mutations** | |
| --- | --- | --- | --- |
|  |  | **surface** | **interior** |
| D_1_R | 1.85e-05 (*) | 145 | 18 |
| D_2_R | 2.94e-06 (*) | 153 | 23 |
| D_3_R | 2.01e-02 (*) | 139 | 44 |
| D_4_R | 1.15e-01 () | 251 | 102 |
| D_5_R | 6.61e-01 () | 272 | 101 |
| 5-HT_2A_R | 3.14e-03 (*) | 155 | 33 |
| 5-HT_2B_R | 3.89e-01 () | 213 | 73 |
| 5-HT_2C_R | 3.13e-05 (*) | 130 | 22 |
| 5-HT_6_R | 1.05e-01 () | 221 | 66 |
| α_1A_-adrenoceptor | 3.61e-01 () | 235 | 74 |
| α_1B_-adrenoceptor | 4.63e-03 (*) | 203 | 33 |
| α_1D_-adrenoceptor | 6.80e-01 () | 274 | 68 |
| α_2A_-adrenoceptor | 3.15e-01 () | 151 | 46 |
| α_2B_-adrenoceptor | 7.74e-02 () | 166 | 47 |
| α_2C_-adrenoceptor | 4.82e-02 (*) | 155 | 44 |
| β_1_-adrenoceptor | 4.91e-01 () | 140 | 48 |
| β_2_-adrenoceptor | 6.27e-03 (*) | 146 | 45 |
| β_3_-adrenoceptor | 8.18e-02 () | 178 | 65 |
| TA_1_R | 8.55e-01 () | 135 | 82 |
| TA_2_R | 2.27e-01 () | 133 | 78 |
| TA_3_R | - | - | - |
| TA_5_R | 2.06e-01 () | 141 | 113 |
| TA_6_R | 3.15e-01 () | 170 | 91 |
| TA_8_R | 6.10e-01 () | 131 | 90 |
| TA_9_R | 3.67e-01 () | 138 | 97 |

**Supplementary data 2: Mutation distribution over Transmembrane domains**


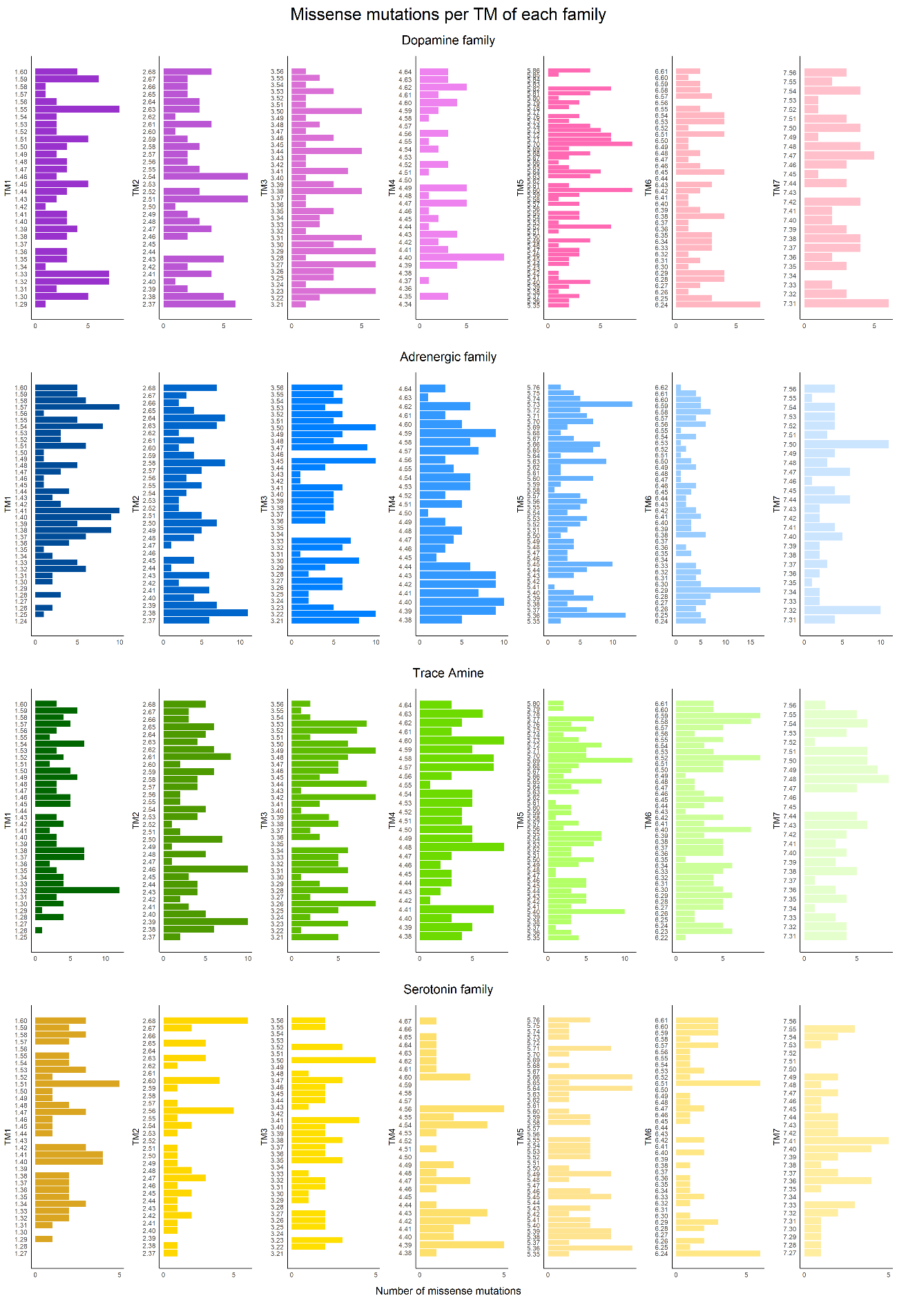


**Fig. A.1.** Distribution of the missense mutations in TM regions of each family (dopamine, serotonin, adrenergic and trace amine receptors). The number of missense variants for each residue of TMs, associated with the Ballesteros-Weinstein (BW) nomenclature. Mutation distribution for the seven transmembrane domains.

### **Supplementary data 3: Relevant residues and missense mutations associated with age**

**Supplementary data 4: Pathogenicity of mutations in relevant residues**

**Table A.2.** Results of the employed pathogenicity prediction programs for the 12 homozygous mutations identified.

| **Receptor** | **Mutation** | **BW** | **Allele frequency** | **MutaFrame** | **SIFT** | **SuSPect** | **POLYPHEN-2** | **SNPs&GO** | **FATHMM** | **PROVEAN** | **PANTHER** | **SNAP2** | **Majority vote** | **Relevant Residue** |
| --- | --- | --- | --- | --- | --- | --- | --- | --- | --- | --- | --- | --- | --- | --- |
| D_1_R | Ser259Tyr |  | 4.13e-3 | Benign | Benign | Benign | Pathogenic | Pathogenic | Benign | Pathogenic | Pathogenic | Pathogenic | **Pathogenic** | GPCR-G-protein interaction; GPCR-Arr-s interaction |
| D_3_R | Arg323Gln | 6.29 | 2.12e-5 | Benign | Benign | Benign | Pathogenic | Benign | Benign | Benign | Pathogenic | Pathogenic | **Benign** | GPCR-G-protein interaction; GPCR-Arr-s interaction |
|  | Val136Ile | 34.51 | 5.09e-4 | Benign | Pathogenic | Benign | Pathogenic | Benign | Benign | Benign | Pathogenic | Benign | **Benign** | GPCR-Arr-s interaction |
| D_5_R | Met75Thr | 2.38 | 8.93e-4 | Benign | Pathogenic | Benign | Pathogenic | Benign | Benign | Benign | Benign | Pathogenic | **Benign** | GPCR-Arr-s interaction |
| β_2_-adrenoceptor | Asn69Ser | 2.4 | 1.15e-3 | Benign | Pathogenic | Pathogenic | Pathogenic | Pathogenic | Benign | Pathogenic | Pathogenic | Pathogenic | **Pathogenic** | Allosteric binding pocket |
| TA_1_R | Asn300Lys | 7.49 | 7.71e-4 | Pathogenic | Benign | Pathogenic | Pathogenic | Benign | Pathogenic | Pathogenic | Pathogenic | Pathogenic | **Pathogenic** | Know activating microdomains |
|  | Ile104Val | 3.33 | 2.66e-4 | Benign | Pathogenic | Benign | Pathogenic | Benign | Benign | Benign | Pathogenic | Benign | **Benign** | Ligand binding pocket |
| TA_5_R | Asp114Val | 3.32 | 3.67e-3 | Benign | Pathogenic | Benign | Pathogenic | Pathogenic | Benign | Pathogenic | Pathogenic | Pathogenic | **Pathogenic** | Ligand binding pocket; Other residues |
| TA_6_R | Thr93Ala | 2.65 | 5.61e-4 | Benign | Pathogenic | Benign | Benign | Benign | Benign | Benign | Benign | Pathogenic | **Benign** | Ligand binding pocket |
|  | Asp281Ala | 6.58 | 1.59e-5 | Benign | Pathogenic | Benign | Benign | Benign | Benign | Pathogenic | Pathogenic | Pathogenic | **Benign** | Ligand binding pocket; Other residues |
|  | Cys291Tyr | 7.33 | 6.43e-2 | Benign | Benign | Benign | Benign | Benign | Benign | Benign | Benign | Pathogenic | **Benign** | Ligand binding pocket |
| TA_8_R | Asp276Ala | 6.54 | 5.81e-4 | Benign | Benign | Benign | Benign | Benign | Benign | Benign | Pathogenic | Pathogenic | **Benign** | Ligand binding pocket; Other residues |
